# The complete chloroplast genome of *Tripsacum laxum* (Gramineae)

**DOI:** 10.1080/23802359.2021.1899872

**Published:** 2021-10-15

**Authors:** Lin Luo, Hui Lin, Hai-lin Luo, Qiao-qi Li, Dong-mei Lin, Guo-Dong Lu

**Affiliations:** National Engineering Research Center of Juncao, Fujian Agriculture and Forestry University, Fuzhou, China

**Keywords:** *Tripsacum laxum*, chloroplast genome, phylogenetic analysis

## Abstract

*Tripsacum laxum* (Guatemalan grass) is a perennial fodder grasses, which is commonly growing in large parts of Africa for a source of livestock feed. It has a high economic value as a forage. In this study, we obtained a complete chloroplast genome of *T. laxum* by Illumina sequencing. The results showed a circular genome of 140,556 bp, including the large single copy region (LSC, 82,939 bp), the small single-copy region (SSC, 12,573 bp), and a pair of 22,522 bp inverted repeat regions (IRs). The circular genome contained 120 genes, including 74 protein-coding genes, eight ribosomal RNA genes and 38 tRNA genes. Evolutionary relationship analysis indicates that *T. laxum* is more closely related to previously reported *T. dactyloides*.

*Tripsacum laxum* (Guatemalan grass) is a perennial fodder grasses, which belongs to the genus *Tripsacum* in Gramineae (http://www.iplant.cn/info/Tripsacum). This species is commonly growing in large parts of Africa for a source of livestock feed (Mwakha 1972; Topps 1986). *T. laxum* with a good adaptation can be tolerant in drought and acid, that is widely introduced as forage in other tropical regions of the world (Compere 1960; Paul et al. 2016). In recent years, *T. laxum* has also been cultivated in Southern China. Previous studies have demonstrated that it is effective for reducing run-off and soil loss (Madhu et al. 2011). The grass has important research significance and application value in the future. Hence, *T. laxum* is need to be better studied and prioritized as a considerable exploitation goal. In this study, we reported the chloroplast (cp) genome of *T. laxum*, which provided important genetic sources for molecular studying.

The fresh leaves were collected from Fujian Agriculture and Forestry University (Fuzhou, China; 26°5′16.04″N, 119°14′12.93″E). The voucher specimen of *T. laxum* was deposited in the National Engineering Research Center of Juncao Technology, Fujian Agriculture and Forestry University with specimen code FJ0623-TL01. The cp complete genomic DNA was extracted using a DNasecure Plant Kit (DP350-03) (TIANGEN, China). Therefore, we used the Illumina high-throughput sequencing to obtain the cp complete genome of *T*. *laxum*. The paired-end clean data were assembled by GetOrganelle kit (version 1.7.2) (Jin et al. 2020). The Geneious R21.0.1 (http://www.geneious.com/, Kearse et al. 2012) software was adopted to annotate the cp genome, while *T. dactyloides* (NC 037087.1) as reference sequences. Then, submitted to GenBank (accession number: MW387499).

The chloroplast sequence of *T. laxum* was 140,556 bp, including the large single-copy region (LSC, 82,939 bp), the small single-copy region (SSC, 12,573 bp), and a pair of 22,522 bp inverted repeat regions (IRs). The circular genome contained 120 genes, including 74 protein-coding genes (68 PCG species), eight ribosomal RNA genes (4 rRNA species) and 38 tRNA genes (21 tRNA species). The overall nucleotide composition of chloroplast genome is: 43,333 bp A (30.8%), 43,210 bp T (30.7%), 26,929 bp C (19.2%), 27,084 bp G (19.3%), and the total GC content of 38.4%.

The sequences alignments with MAFFT v7.304 (Katoh and Standley 2013) and MEGAX (Kumar et al. 2018) software was used to construct a maximum likelihood (ML) tree with 1000 bootstrap replications. The ML method based on 15 published complete chloroplast genome sequences which were downloaded from NCBI GenBank database, and constructed a phylogenetic tree (Figure 1) to confirm the relationship between *T. laxum* and other related taxa, while *Oryza rufipogon*, *Oryza sativa* were used as outgroups.

**Figure 1.**
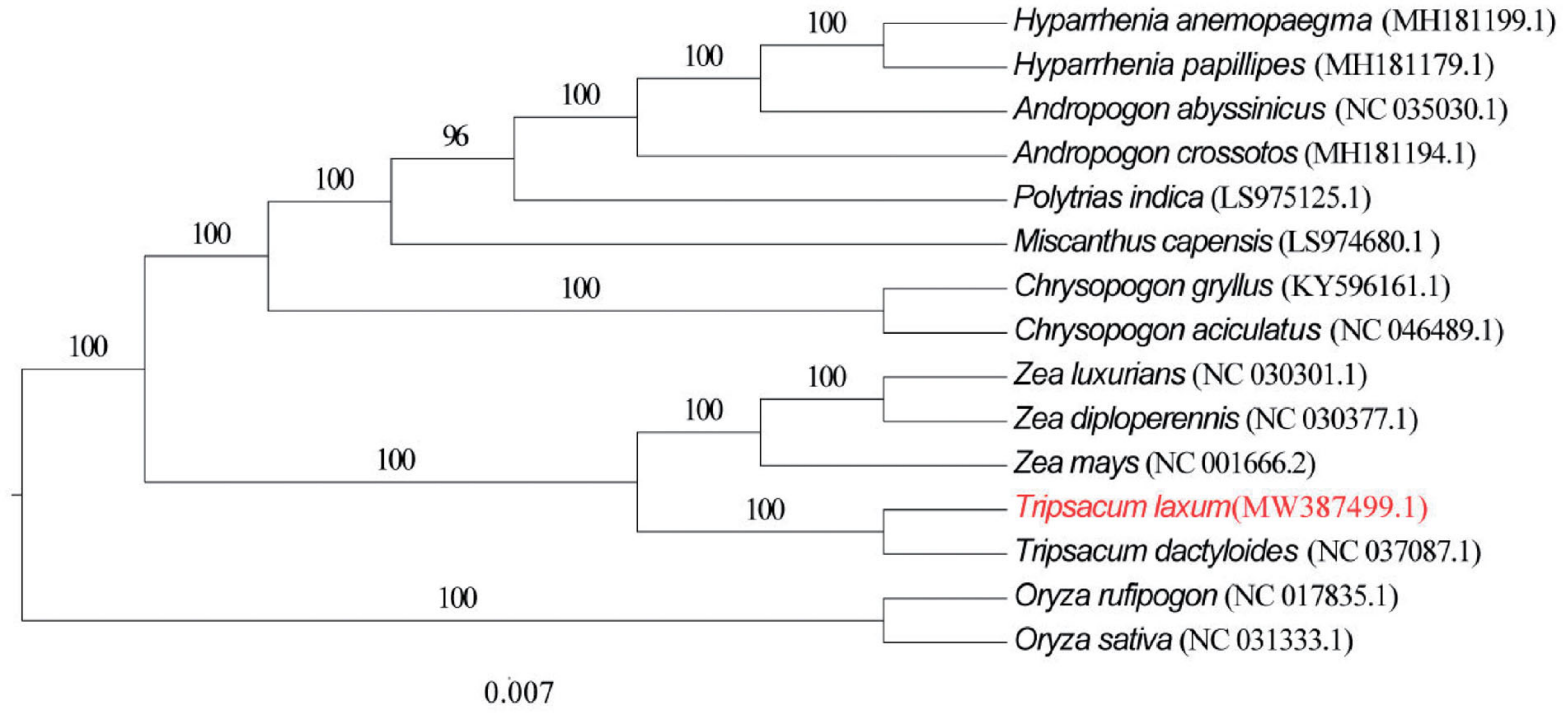
The maximum likelihood (ML) phylogenetic tree based on 15 complete chloroplast genomes, and *Oryza rufipogon*, *Oryza sativa were used* as outgroups.

Phylogenomic analysis was conducted by RAxML v.8.2.8 (Stamatakis 2014). The ML phylogenetic analysis showed that *T. laxum* is closely related to *T. dactyloides* (NC 037087.1) in Gramineae (Figure 1).

## Data Availability

The data that support the findings at this study have been stored in the NCBI database under accession number of MW387499 (https://www.ncbi.nlm.nih.gov/) and the NCBI SRA (BioProject: PRJNA688126) accession number of SRR13311453 (https://www.ncbi.nlm.nih.gov/sra).

## References

[CIT0001] Compere R. 1960. Introduction into Kivu (Congo) of *T. laxum*, a forage plant for dairy cows in the dry season. Bulletin Agricole du Congo Belge. 51(5):1085–1103.

[CIT0003] Jin JJ, Yu WB, Yang JB, Song Y, Depamphilis CW, Yi TS, Li DZ. 2020. GetOrganelle: a fast and versatile toolkit for accurate de novo assembly of organelle genomes. Genome Biol. 21(1):241 32912315 10.1186/s13059-020-02154-5 PMC7488116

[CIT0004] Katoh K, Standley DM. 2013. MAFFT multiple sequence alignment software version 7: improvements in performance and usability. Mol Biol Evol. 30(4):772–780. 23329690 10.1093/molbev/mst010 PMC3603318

[CIT0005] Kearse M, Moir R, Wilson A, Stones-Havas S, Cheung M, Sturrock S, Buxton S, Cooper A, Markowitz S, Duran C, et al. 2012. Geneious basic: an integrated and extendable desktop software platform for the organization and analysis of sequence data. Bioinformatics. 28(12):1647–1649. 22543367 10.1093/bioinformatics/bts199 PMC3371832

[CIT0006] Kumar S, Stecher G, Li M, Knyaz C, Tamura K. 2018. MEGA X: molecular evolutionary genetics analysis across computing platforms. Mol Biol Evol. 35(6):1547–1549. 29722887 10.1093/molbev/msy096 PMC5967553

[CIT0007] Madhu M, Raghunath B, Tripathi KP, Sam MJ, Chandran B, Mohanraj R, Haldorai B. 2011. Vegetative barrier with contour staggered trenches for resource conservation in new tea plantations of the Nilgiris. Indian J Soil Conserv. 39(1):33–36.

[CIT0008] Mwakha E. 1972. Effect of cutting frequency on productivity of Napier and Guatemala grasses in Western Kenya. East African Agri Forest J. 37(3):206–210.

[CIT0009] Paul BK, Muhimuzi FL, Bacigale SB, Benjamin MMW, Chiuri WL, Amzati GS, Maass BL. 2016. Towards an assessment of on-farm niches for improved forages in Sud-Kivu, DR Congo. J Agri Rural Deve Tropics Subtropics. 117(2):243–254.

[CIT0010] Stamatakis A. 2014. RAxML version 8: a tool for phylogenetic analysis and post-analysis of large phylogenies. Bioinformatics. 30(9):1312–1313. 24451623 10.1093/bioinformatics/btu033 PMC3998144

[CIT0011] Topps JH. 1986. Pasture improvement research in Eastern and Southern Africa: Proceedings of a Workshop held in Harare, Zimbabwe, 17–21 September, 1984. Edited by Jackson A. Kategile. Intl Development Res. Centre, Ottawa, Canada. Agric Wastes. 17(2):157–158.

